# Physical activity and sport practice to improve balance control of visually impaired individuals: a narrative review with future perspectives

**DOI:** 10.3389/fspor.2023.1260942

**Published:** 2023-09-13

**Authors:** Giuditta Carretti, Mirko Manetti, Mirca Marini

**Affiliations:** Section of Anatomy and Histology, Department of Experimental and Clinical Medicine, University of Florence, Florence, Italy

**Keywords:** visual disability, adapted physical activity, sport practice, balance training, postural control, exercise specialist

## Abstract

Visual disability negatively impacts balance, everyday self-efficacy, and mobility and often leads affected subjects to perceive physical exercise as a burdensome challenge thus discouraging them from practicing. Despite the well-proven benefits of regular physical activity in visually impaired people, especially addressing postural control, there are no specific guidelines and most of the available literature seems to be flawed by critical issues. Given the wide heterogeneity and the multidimensional needs of this population, a more realistic and target-specific perspective is needed in order to properly investigate and promote exercise practice and adherence for balance improvement. On this basis, through a critical overview of the recent literature, the present article aimed to enrich the current knowledge about this topic by providing innovative suggestions, both practical and methodological, and specifically deepening the disability-related deficits and peculiarities of different age ranges. Moreover, since a multidisciplinary approach is advisable when designing and leading exercise protocols tailored to visually impaired individuals, such innovative hints also highlighted the central role of the adapted physical activity specialist, hence contributing to foster its official professional recognition and involvement in this field.

## Introduction

Global health is currently jeopardized by three main trends, precisely, ageing population, globalization, and technological advance all of which promoting unhealthy behaviors and frames. Inevitably, the growing prevalence of chronic diseases and their risk factors is rapidly becoming a concerning global issue (1, 2). Physical inactivity, currently identified as the fourth leading risk factor for global mortality, is rising worldwide thus negatively affecting general health of all ages population (3–7). Sedentary lifestyle is associated with decreased quality of life and physical functioning, both causing socio-economic and psychophysical burdens on public health and individual daily life (8, 9). In an aging society, visual impairment is an increasingly prevalent condition especially in developed countries because of the uprising incidence of age-related eye diseases and diabetic retinopathy (10–13). Age regardless, vision impairment has been found to be strongly linked with lower everyday self-efficacy/functioning, both psychologically and physically (14–17) when compared with sighted peers (18–20). In particular, given the disability-related balance and postural control deficit (21–24), visually impaired (VI) individuals show higher risk of falls and accidental injuries (19, 25, 26), therefore perceiving exercise as an overwhelming challenge. Such conditions deeply impact autonomy, social interaction, overall wellbeing, and leisure/sport activity participation (27–32). Despite the well-known benefits of regular physical activity in disabled subjects (33–35), no VI-specific directives are available, and a large proportion of VI individuals does not meet the daily movement guidelines established for the general population (15, 36–39). Fear of falling and postural/proprioceptive control deficiencies play a crucial role in such lack of exercise, frequently leading those individuals to avoid any recreative or sportive physical engagement opportunity (12, 16, 20, 22, 28).

Balance control is the result of an orchestrated integration of visual, vestibular, and proprioceptive input, and deeply affects static and dynamic posture, both in daily life activities and in the recreative/sportive ones (40–43). When alterations occur in even one of those balance-related systems, psychophysical disorders, disabilities, loss of autonomy and functionality inevitably onset (14, 44). It has been widely demonstrated, in all age groups, that regular physical activity improves balance by stimulating proprioceptive postural control, general and segmental coordination, strength, and reaction time (41, 45). Despite the extensive scientific evidence, most studies focused on elderly fall prevention or post-injury and pathological frames rehabilitation, while just a few investigated balance training protocols specifically addressed to VI subjects (10, 46–52). Actually, regarding this target group, many studies deepened the topic more in a social inclusion than in a functional and performative perspective (53–56). Moreover, in case of visual disability, given the high percentage of aging-related onset, the wide range of visual deficit, and the safety purposed need of working out in small class, research often struggled to provide an exhaustive overview of the more effective methodological approaches for this variegated population (57). Visual impairment term includes a broad spectrum of etiology, time of onset and severity level which significantly affect subjective peculiarities and needs (58, 59). In order to provide a global and safe management of such heterogeneity, a multidimensional methodological approach might be preferred. Specifically regarding the training field, protocols should involve both collective and individual sessions, which should be designed, leaded and monitored by an adapted exercise specialist (60, 61).

Given the proved weight of postural control on health and quality of life, especially in case of visual disability (27), an updated review of the current knowledge, enriched with an evidence-based overview of the most innovative tools and technologies, might help designing easily applicable and age-tailored protocols for the VI population.

## Specific aims and methods

On the aforementioned basis, the present narrative review aimed to offer not only a comprehensive summary of the recent literature investigating balance training for VI subjects but also innovative cues for future applications in that field. In detail, current balance training methodologies tailored to this target population were deepened in an age range-perspective aiming to provide a specific focus without losing the overall view. Indeed, presenting and discussing, side by side, the current research findings for each age group may ease to identify specific needs and balance sensitive/critical periods, thus guiding and optimizing field-specific investments. Hopefully, such perspective might also provide methodological tools to boost protocol adherence and effectiveness, together with a growing awareness of the central role of the adapted physical activity specialist. Concerning the applied criteria to source the investigated literature, a multiple database search (Pubmed, Web of Science and Scopus) was performed. Specifically, English language papers published from 2000 to 2023 have been found using keywords and sentences such as “visual disability”, “physical activity intervention for visually impaired”, “visually impaired balance”, “balance training and visual impairment”, “postural control in blind subjects”, “adapted sport and visual disability”, and subjectively prioritizing recent investigations and innovative methodological approaches/tools. Moreover, drawn from the identified articles bibliography and using the “similar articles” suggestions provided by scientific database, further literature fulfilling the abovementioned subjective criteria was selected.

## Visual impairment implications on postural control

Congenital or acquired visual disability leads to psychophysical development delays and motor pattern alterations that consequently affect postural control (62–64). Posture, strongly linked to stability, balance and functionality, is a multidimensional concept able to impact daily life activities, social interaction, autonomy, and quality of life (65, 66). Motor behaviors, either purposeful or involuntary, are characterized by a bidirectional interplay between the body and the surrounding environment (19, 67). Such inevitable interaction is primarily influenced and driven by visual input, thus giving to this sensory system a crucial role in postural control and adjustments (68–72). Vision is indispensable to provide instant information regarding body-space interaction, movement precision/orientation and motor action timing. Visual impairment often isolates subjects from the external environment thus depriving them of the sensorimotor feedback needed for functional body mechanics acquisition and effective development of postural reflexes (64). The loss of the aforementioned feedback results in postural deviations chiefly characterized by backward leaning trunk, increased dorsal kyphosis, dropped shoulders, head forward compensating position, and valgus flat feet (63, 73, 74). All these anatomo-functional abnormalities, added to uncoordinated limb movements, decreased gait speed, spatial orientation difficulties and body image alteration, lead to faulty motor patterns and dynamic balance control issues (75–78).

Since posture turned out to be a psychosomatic affair, visual impairment can negatively impact not only motricity but also educational and social growth, thus feeding a dangerous vicious cycle (79). Blind children, due to disability-related development delays, exhibit poor body language, and ineffective facial expressions, gestures, and communication (31, 80). Though knowing sport and leisure activities benefits, overprotective parents often prevent them to experience those formative occasions (81–83). Unfortunately, postural control, both in terms of perception and execution, cannot be learnt and mastered without a constant interaction with others in a real environmental context. This lack of learning and peer-interacting opportunities, in addition to boosting fear and frustration, deeply affects postural behaviors (68, 84, 85).

Postural stability is referred to the body skill of maintaining balance and it is often assessed through postural sway analysis and quantification (86, 87). According to research findings, visual disability-affected individuals generally show increased postural sways hence experiencing higher fear of falling (88, 89). Such evaluation tool can help outlining a postural profile in semi-static and study setting, but it is fundamental to remind that human postural control is predominantly motion and reality connected (90). Therefore, balance control of VI individuals should be investigated not only in a fall prevention perspective, but through a comprehensive analysis of the main motor pattern performance in daily life frames, taking into account the disability-related alterations and compensative strategies (91, 92).

## Adapted physical activity benefits on balance control in visually impaired individuals

Fundamental motor skills play a key role in learning/development of complex gestures required to effectively perform daily life activities and participate in specific physical activity and sport contexts. Among them, balance and stability skills, both static and dynamic, have the power to influence the correct structuring of motor competence as well as physical fitness level and psychophysical health (93–95).

It has been recently demonstrated that children motor competence perception is more impacting than actual motor abilities on their overall fitness level (96–99). During childhood, physical activity promotes motor skill development and, before self-awareness acquisition, children practice it despite their real competence and results, thus unintentionally increasing motor learning opportunities. After that stage, detectable at about the age of eight, a vicious spiral of physical activity disengagement onsets in children showing low motor competence (100–103). Literature has frequently reported that VI children and adolescents, when compared to sighted peers, tend to conduct a more sedentary life and to exhibit lower physical fitness (20, 31, 62, 104–108). This latter, along with a coherent motor skills impairment, seems to arise mostly from low participation in physical and after-school sport activities (37, 94, 109–111). Similarly, concerns regarding instructor methodological competence, environmental safety, lack of support, convenience and mobility often lead VI adults to not engage in physical/sport activities (112–115). Several studies reported that lower postural stability of VI individuals, compared to sighted ones, is due to the absence of natural compensatory mechanisms based on enhanced non-visual input use for balance control (91, 116–118).

Considering the elderly population, research mainly investigated balance control and fall prevention in healthy subjects affected by age-related visual dysfunctions. Conversely, only a few studies addressed visually disabled individuals highlighting that multimodal exercises can improve their postural control (119, 120). Accordingly, it has been suggested that balance control improvement in sight impaired people requires a conscious behavioral compensation achievable through a targeted training involving balance and navigation skills (121, 122). Based on this evidence, recent research highlighted a positive link between habitual physical activity levels and balance performance in those subjects, as briefly summarized in Table 1. In fact, blind individuals regularly practicing physical exercise show more functional gait pattern and perform better in balance and navigation tasks than sedentary peers (111, 122, 148). Precisely, it has been shown that a 12-week specific training protocol can significantly increase blind adults balance performance, thus confirming the effectiveness of adapted physical activity on their postural control and everyday mobility enhancement (149). Regarding VI children and adolescents, a further study detected that higher amount of physical exercise was deeply related to a postural sway decrease and an improvement in single-leg stance time, orientation abilities, and dynamic gait stability (123, 128). Since balance skills and spatial cognitive functions are development-dependent, the interrelation between motor activity and postural control should be especially promoted during childhood and adolescence (132, 133). Exploiting such learning-sensitive phase, the onset of disability-related balance deficit, postural alterations, mobility issues and fall fear could be effectively prevented or counteracted through a ludic, active, and challenging approach. Despite that, there is an evident lack of literature concerning the most effective exercise types, duration and methodologies in the visually disabled population (129). Since these individuals mostly rely on proprioceptive and vestibular input for postural control, to date, it seems that training protocols promoting such vicariant sense recruitment may be more effective (27, 71, 127, 137, 150, 151).

**Table 1:** Summary of current knowledge (2000–2023) and future perspectives of balance training for visually impaired individuals.

Investigated age range	Main objectives	Applied methodologies (References)	Demonstrated benefits	Highlighted critical issues (References)	Future perspectives (References)
Children and youth (6–12 years old)	Inclusiveness, general physical fitness, balance, and coordination	Recreational physical activity (123–125), Yoga (126), Dance and Pilates (127)	Psychophysical health, social interaction, autonomy and self-esteem, correct structuring of motor competence, orientation abilities, dynamic gait stability	No specific guidelines, small sample size, protocol shortness, no exercise specialist leading, insufficient family involvement (128–131)	Early proprioceptive training, school-based protocol integration, holistic psychophysical engagement, graduated adapted physical activity specialist involvement (27, 61, 127, 132)
Adolescents and young adults (13–30 years old)	Social integration, general physical fitness, healthy lifestyle promotion	Leisure and general physical activity (133, 134), rope jumping (112)	Fall fear prevention, psychophysical well-being, orientation abilities, dynamic gait stability, single-leg stance time, coordination	No specific guidelines, mall sample size, protocol shortness, no ludic/enjoyable approach, insufficient socio-economic support to families, no adapted exercise specialist leading (54, 128, 130, 135)	Autonomous urban mobility facilitation, blind sport promotion, technological tools, multimodal proprioceptive training, holistic psychophysical engagement, graduated adapted physical activity specialist involvement (27, 56, 61, 136)
Over 50 adults and elderly	Fall prevention, daily life self-efficacy, general health and successful aging promotion, inclusiveness	General balance training and Otago exercise program (60, 119, 120, 122, 137, 138), Tai-Chi (46), Yoga (139), Pilates (140), Dance (27, 49, 141)	Self-efficacy in daily life activities, autonomy, psychophysical well-being and quality of life, postural control, functional gait patterns, navigation skills	Small sample size, age/gender imbalance, protocol shortness, facilities accessibility, no adapted exercise specialist leading, sanitary approach (28, 131, 142–145)	Core stability training, multimodal proprioceptive training, quantitative assessment of functional parameters through wearable devices, virtual reality training, blind sport promotion, graduated adapted physical activity specialist involvement (27, 54, 61, 146, 147)

## Evidence-based training methodologies and sport activities

**Figure 1:**
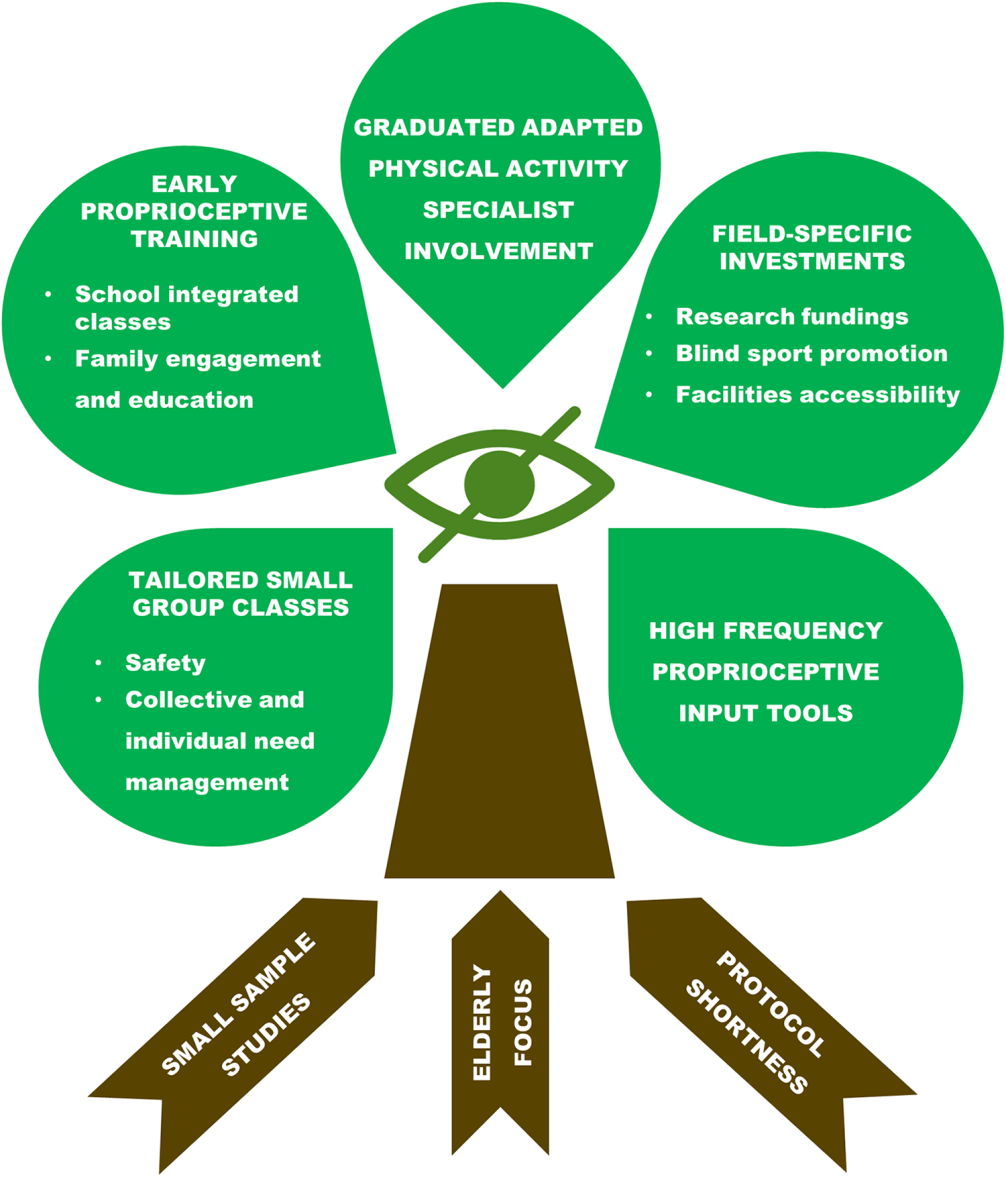
Tree diagram of the evidence-based critical issues (brown labels on tree roots) and the related applicable suggestions (green labels on tree crown) to improve balance control of visually impaired individuals through physical activity and sport practice.

Literature specifically addressing balance training for VI people is scarce and presents critical issues often attributed to research design weaknesses or errors, as reported in recent reviews (130, 142, 152). The main intrinsic issues concern the small sample size investigated and the consequent statistical reliability, as well as gender and age imbalance showing a clear predominance of female and elderly participation. In addition, there are almost none quantitative evaluation tools validated for this target population (57), and most of the studies last 8–12 weeks or less, thus being supposed to limit exercise psychophysical benefits and their maintenance over time (131) (Figure 1). Actually, if analyzed in a target-specific perspective, those potential limitations frequently reflect the peculiar multidimensional and safety needs of this heterogeneous population. In particular, adapted physical activity protocols tailored to VI subjects should be conceived, leaded and carried on in small groups thus granting collective and individual support, assistance, and safety (61). Regarding overrepresentation of female and elderly participants it must be considered that such proportion simply reflects the real socio-demographic characteristics of the worldwide population affected by visual impairment (58, 153). Finally, concerning protocols duration, it has been demonstrated that balance improvement is more affected by the frequency and peculiarities of the proprioceptive input applied than the intervention length (154, 155). Honestly, targeted physical exercise benefits maintenance, especially in disabled subjects, strongly relies on constant practice over time (156, 157). However, this criticality should not be imputed to study design weakness but more to the scarcity of field-specific investments in term of research funds, blind sports promotion, facilities accessibility, and involvement of adapted physical activity graduated specialists (28, 143–145) (Figure 1). Considering this necessary premise, current evidence emerged from interventions aimed to balance improvement in VI individuals are hereafter reported and concisely summarized in Table 1. Several studies were fall prevention aimed and, hence, they frequently addressed elderly and over fifty subject sample. As far as the applied methodology is concerned, some interventions used general balance training protocols while others opted for a validated exercise program such as Otago (60, 119, 120, 122, 138, 151). Additionally, recent evidence about Tai-Chi, yoga, Pilates and dance benefits on VI adults and elderly balance highlighted the relevance of a holistic involvement of this target population (27, 46, 49, 139–141). Focusing on research addressing balance improvement of young subjects affected by visual impairment, there is no univocal evidence about preferable or more effective methodologies and activities to apply (128). Therefore, current literature ranges from general physical activity protocols, even school integrated, to coordinative exercise such as rope jumping and holistic disciplines like yoga, dance, and Pilates (112, 124–127, 134). Since balance skills are development sensitive and the perception of motor competences, as well as family support, can deeply impact exercise engagement, adapted sports for VI children and adolescents should become a socio-economic and educational investment priority (56, 136, 158, 159). Finally, there is a variegated body of literature investigating athletes affected by visual impairment, both amateur or competitive, which considered and managed balance control as a crucial sport performance prerequisite (77, 160–169). Given the essential link between dynamic balance, anatomo-functional prerequisites and their on-field/in-game adaptation, it is widely believed that these targeted interventions should be conducted respecting and recalling the real sport specific frame (61).

## Future perspectives and innovations

Although the well documented cause-effect relationship between physical inactivity and overall health parameters maintenance, balance control included, little is still known about visual impairment tailored exercise (170). Recent innovative approaches focused on core stability training, unstable surface utilization and multimodal proprioceptive input have reported findings worthy to be deepened (49, 171–174). At the same time, it is available in literature a rising application of virtual reality and technological tools, like wearable devices, able to monitor physical/functional parameters or to provide haptic and vibration feedback aimed to balance training of VI individuals (146, 147, 175, 176). Despite those few pioneering interventions, the main critical issues are related to the lack of guideline and literature investigating exercise effectiveness in a dose-response perspective on this target population. Indeed, almost all research applied low-intensity physical activities thus frequently overlooking the crucial link between fitness, motor competence perception and anatomo-functional parameters such as postural control (142). In future investigations, though focusing on balance and stability, it should be recommended to integrate such aimed protocols with moderate and vigorous intensity physical activities, hence globally affecting overall fitness and functionality (177). Moreover, there is scarcity of literature investigating the involvement and enjoyment of VI population approaching and consistently carrying on exercise practice (135). Indeed, the complex needs of these subjects require a global management able to consider not only protocols application and effectiveness, but also disability-specific communication and workout leading strategies to make them enjoyable and attractive, ultimately promoting exercise adherence (61, 131, 178). Unfortunately, balance improvement interventions addressing those who are visual impairment affected are often fall prevention oriented or based exclusively on basic daily life activities and mobility training (4, 5, 12, 14, 15, 41, 48, 179, 180). Despite the undeniable importance of the aforementioned aims, such a mostly sanitary/rehabilitative approach risks to discourage visually disabled individuals, especially youth, to perceive physical exercise as pleasant and worthy of engagement. On the basis of the rising recognition of health-related disparities experienced and reported by disabled people, it becomes crucial to grant them inclusiveness without losing sight both of their integration with healthy population and their peculiar needs (54). Therefore, given the well-known holistic involvement of disabled subjects during exercise practice and the acquired multidisciplinary competences of the graduated adapted physical activity specialists, it is advisable that they become the official professionals operating in such a sensitive field.

## Data Availability

The original contributions presented in the study are included in the article/Supplementary Material, further inquiries can be directed to the corresponding author.
